# Dielectric Constant Estimation of Spherical Particle-Filled Nanocomposites Based on the Poon and Shin Model, Considering Interphase Properties

**DOI:** 10.3390/polym17081035

**Published:** 2025-04-11

**Authors:** Bin Tang, Xue Liu, Shengxiang Deng, Wei Zhong, Jiang Shao

**Affiliations:** 1 School of Mechanical Engineering, Jiangsu University of Science and Technology, Zhenjiang 212100, China; tangbin29@21cn.com; 2 The School of Optoelectronic Manufacturing, Zhejiang Industry and Trade Vocational College, Wenzhou 325002, China; 3 Henan Key Laboratory of Polyoxometalate Chemistry, College of Chemistry and Chemical Engineering, Henan University, Kaifeng 475004, China; 4 Department of Energy and Power Engineering, School of Mechanical and Automotive Engineering, Shanghai University of Engineering Science, Shanghai 201620, China; csdsx@163.com

**Keywords:** modeling, interphase properties, dielectric constant, polymeric nanocomposite

## Abstract

A revised version of the Poon and Shin (PS) model, incorporating the effects of the interphase, is introduced to predict the dielectric permittivity of polymer nanocomposites reinforced with spherical nanoparticles. In this modified approach, both the spherical nanoparticle and its surrounding interphase region are treated as an equivalent nanoparticle, modeled as a core–shell structure. This assumption enables a more accurate representation of the composite, where the polymer matrix and the equivalent nanoparticles form a homogeneous mixture. The process of calculating the dielectric permittivity of the composite occurs in two distinct steps. Initially, the dielectric permittivity of the equivalent particle—comprising both the nanoparticle core and its interphase—is computed. Subsequently, the overall dielectric permittivity of the composite material is determined, considering the properties of the polymer substrate and the equivalent nanoparticles, all within the framework of the modified PS model. To verify the validity of the proposed model, experimental data are compared against the predicted values, showing a high level of agreement when the interphase characteristics are appropriately incorporated. Additionally, the influence of various factors, including the properties of the spherical nanoparticles, the interphase, and the polymer matrix, on the dielectric performance of the nanocomposite is thoroughly investigated. This enhanced PS model offers a valuable theoretical framework for designing polymer–spherical nanoparticle composites with superior dielectric properties, paving the way for their potential application in advanced electronic and energy storage devices.

## 1. Introduction

In recent years, dielectric polymer materials have gained considerable attention due to their potential applications in a wide range of advanced technologies, particularly in energy storage systems, flexible electronics, and wearable devices [1,2,3,4]. These materials are widely used as substrates in high-performance capacitors, sensors, and transistors due to their lightweight nature, mechanical flexibility, and ease of processing [5,6,7]. Some of the most commonly employed polymer matrices include polyimide (PI) [8], poly(vinylidene fluoride) (PVDF) [9], and polydimethylsiloxane (PDMS) [10], among others. Despite their excellent mechanical strength, flexibility, and chemical stability, these polymeric materials typically exhibit low dielectric constants, which significantly hinder their effectiveness in energy storage and electronic applications [11,12]. This limitation has prompted extensive research efforts aimed at enhancing the dielectric properties of polymer-based materials. Strategies such as incorporating high-dielectric nanofillers, modifying polymer structures at the molecular level, and optimizing interfacial interactions have been explored to improve their overall dielectric performance [13,14,15,16]. By overcoming these challenges, dielectric polymers with superior properties can be developed, paving the way for next-generation electronic and energy storage technologies.

One effective approach to improving the dielectric performance of polymer nanocomposites is the incorporation of high-dielectric nanoparticles into polymer matrices. Representative nanoparticles commonly used for this purpose include barium titanate (BT) and aluminum oxide (Al_2_O_3_) [17,18]. For example, Goyal et al. reported the development of BT-filled polymethylmethacrylate (PMMA) composites, which exhibited significantly improved dielectric properties compared to the pure PMMA matrix [19]. The maximum dielectric constant of the BT-PMMA composite reached 69 at a filler content of 65 vol.%. Kim et al. synthesized a high-energy-density poly(vinylidene fluoride-co-hexafluoropropylene) nanocomposite reinforced with BT nanoparticles, achieving a maximum dielectric constant of 35 with 50 vol.% filler content [20]. Zahhaf et al. introduced structured Al_2_O_3_-PDMS composites which demonstrated superior dielectric properties over their unstructured counterparts [21]. The dielectric constant of the structured Al_2_O_3_-PDMS composites increased with the Al_2_O_3_ volume fraction, reaching a dielectric permittivity of 6.25 at an Al_2_O_3_ content of 40 vol.%.

In the nanocomposites reported in the literature, nanofillers are typically dispersed within the polymer matrix, where they are enveloped by the surrounding polymer material. However, due to their inherently high surface area, nanoparticles tend to aggregate rather than remain uniformly separated, a phenomenon that leads to a significant deterioration in both the mechanical and electrical properties of the composite [22,23,24]. To counteract this, various techniques, such as ultrasonication and roll milling, are employed to disrupt these agglomerations and facilitate better dispersion [6,25,26]. Furthermore, an interphase region forms at the interface between the nanofillers and the polymer matrix, arising from the interactions between the two phases [27]. This interphase zone, exhibiting unique properties that distinguish it from both the polymer matrix and the nanofillers, is known to have a profound impact on the overall mechanical and electrical performance of the nanocomposite [28,29,30]. However, the characterization of this interphase region is particularly challenging due to its complex and elusive nature, as well as the limitations of current measurement techniques, which make it difficult to directly assess its physical properties, such as its thickness and dielectric constant. As a result, theoretical methods have become indispensable tools in nanocomposite research, offering invaluable insights for the design and optimization of these materials. Numerous models grounded in distinct theoretical approaches, including the Maxwell–Garnett, Bergman, and Lichtenecker models, have been developed to predict the performance of nanocomposites and guide their fabrication [31,32,33].

**Figure 1:**
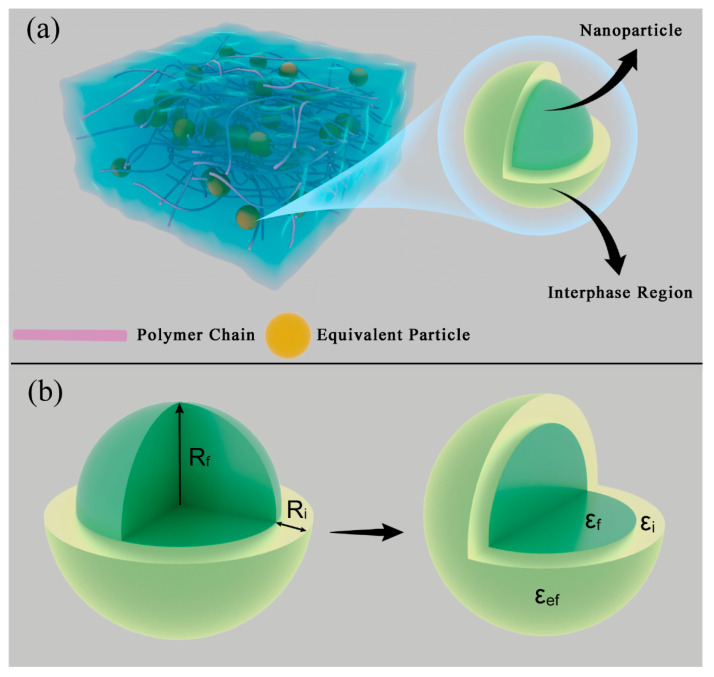
(**a**) Schematic representation of the spherical nanoparticle, its surrounding interphase, and the corresponding equivalent particle model; (**b**) Schematic illustration of the hybrid particle.

Poon and Shin developed a straightforward analytical formula to predict the dielectric constants of particulate-filled composite materials, assuming that the inclusions are uniformly distributed within the matrix [34,35]. However, it is important to note that the PS model does not account for the interphase effects, which can significantly influence the overall properties of the composite. While various models have been developed in previous studies to address core–shell type inclusions [36,37,38,39], research on the intrinsic characteristics of the interphase—such as its thickness and dielectric constant, and their influence on the equivalent particle content and dielectric properties of composites—remains limited. In this study, we extend the PS model to develop a novel mathematical framework that can predict the dielectric constant of spherical particle-filled nanocomposites, while properly incorporating the effects of the interphase. The interphase is modeled as a shell surrounding the spherical particles, as illustrated in Figure 1. The nanocomposite is considered to consist of the polymer matrix, spherical inclusions, and the interphase region. Experimental data are used to validate the accuracy of the modified PS model, with excellent agreement observed between the predicted and measured values. The primary objective of this work is to provide a useful tool for predicting the dielectric properties of polymeric nanocomposites reinforced with spherical inclusions, while simultaneously evaluating the influence of the interphase characteristics.

## 2. Model Description

In the PS model (Equation (1)), the displacement field experienced by a single particle is considered to arise from both the surrounding matrix and the polarization of the inclusions embedded within the matrix. The model also accounts for the interactions between these embedded inclusions. However, as previously noted, the PS model does not incorporate the effects of the interphase. Extensive research has demonstrated that an interphase zone develops at the particle surface [40,41], as depicted in Figure 1a,b. This interphase region represents a modified section of the polymer matrix and is confined solely to the outer layer of the nanoparticle. This interphase is primarily a result of the interactions between the filler particles and the polymer matrix. Additionally, the surface area of the embedded inclusions plays a crucial role in the formation and characteristics of the interphase region.

(1) $$\varepsilon =\varepsilon_{m}+\frac{\left(\varepsilon_{f}-\varepsilon_{m}\right)V_{f}}{\left(1-V_{f}\right)\frac{\varepsilon_{f}+2\varepsilon_{m}-V_{f}(\varepsilon_{f}-\varepsilon_{m})}{3\varepsilon_{m}}+V_{f}}$$

where ε, $\varepsilon_{m}$, and $\varepsilon_{f}$ are the permittivity of composite, permittivity of matrix, and permittivity of particles, respectively. $V_{f}$ is the volume fraction of particle.

Previous research has established a connection between the interfacial properties and both the mechanical and electrical performance of composite materials [42,43]. Specifically, the presence of interphase regions can enhance the effective volume fraction of conductive particles, thereby facilitating the development of conductive pathways and lowering the percolation threshold. To simplify the analysis, this study assumes that the particles are idealized as spherical. To account for the interphase effects, a model of equivalent particles with core–shell structures is proposed, where the interphase region is represented as a shell surrounding the spherical core, as shown in Figure 1. The dielectric permittivity of these equivalent particles is initially determined using the Tanaka formula, which is also known as the Maxwell–Garnett formula, as presented in Equation (2) [27,44].

(2) $$\varepsilon_{H}=\varepsilon_{s}\frac{\varepsilon_{c}\left(1+2\rho \right)+2\varepsilon_{s}(1-\rho )}{\varepsilon_{c}\left(1-\rho \right)+\varepsilon_{s}(2+\rho )}$$

where $\varepsilon_{H}$, $\varepsilon_{c}$, and $\varepsilon_{s}$ denote permittivity of the hybrid particle, permittivity of the core part, and permittivity of the shell, respectively. In the Tanaka formula, the parameter ρ is dominated by the size of inner part (radius, R) and shell (thickness, $R_{s}$), as defined in Equation (3).

(3) $$\rho =\frac{R^{3}}{{\left(R+R_{s}\right)}^{3}}$$

The Tanaka formula was initially developed to determine the dielectric constant of hybrid particles with core–shell configurations. In the present study, the spherical particle and its surrounding interphase are treated as equivalent particles with a core–shell structure. In this model, the spherical particle and the interphase region correspond to the core and shell components, respectively, as defined in the Tanaka formula. Consequently, the dielectric constant of the equivalent particle, which includes both the spherical particle and the associated interphase, is expressed as follows:

(4) $$\varepsilon_{ef}=\varepsilon_{i}\frac{\varepsilon_{f}\left(1+2\rho^{\prime }\right)+2\varepsilon_{i}(1-\rho^{\prime })}{\varepsilon_{f}\left(1-\rho^{\prime }\right)+\varepsilon_{i}(2+\rho^{\prime })}$$

In this expression, $\varepsilon_{ef}$ denotes the dielectric permittivity of the equivalent particles, while $\varepsilon_{f}$ refers to the permittivity of the inclusions. The dielectric constant of the shell-like interphase is represented by $\varepsilon_{i}$. Additionally, $\rho^{\prime }$ is defined by Equation (5), where $R_{f}$ and $R_{i}$ represent the radius of the inclusions and the thickness of the interphase, respectively.

(5) $$\rho^{\prime }=\frac{{R_{f}}^{3}}{{\left(R_{f}+R_{i}\right)}^{3}}$$

The concentration of particles plays a crucial role in determining the dielectric permittivity of the nanocomposite. Given that the interphase region enhances the effective concentration of the filler particles, it is essential to calculate the volume fraction of the equivalent nanoparticles, denoted as $V_{ef}$. The theoretical filler concentration, represented by $V_{f}$, is defined as the ratio of the total volume of the inclusions ($V_{p}$) to the volume of the nanocomposite ($V_{n}$), as shown in Equation (6).

(6) $$V_{f}=\frac{V_{p}}{V_{n}}$$

where $V_{p}$ is calculated by the following formula:

(7) $$V_{p}=n\nu_{p}$$

In Equation (7), n represents the total number of filler particles, while $V_{p}$ refers to the volume of an individual particle. Since the particles in this study are modeled as spheres, the volume of a single spherical particle $V_{p}$ is calculated according to Equation (8):

(8) $$\nu_{p}=\frac{4}{3}\pi {R_{f}}^{3}$$

The volume of an equivalent particle is determined by adding the volume of the filler particle $V_{p}$ and the volume of the associated interphase region $\nu_{i}$. Consequently, the total volume of a single equivalent particle $\nu_{ef}$ is given by the following expression:

(9) $$\nu_{ef}=\nu_{p}+\nu_{i}=\frac{4}{3}\pi {\left(R_{f}+R_{i}\right)}^{3}$$

According to Equations (5), (8) and (9), the relationship between the volume of single spherical particle $\nu_{p}$ and volume of single equivalent particle $\nu_{ef}$ can be established as shown in Equation (10).

(10) $$\nu_{ef}=\frac{\nu_{p}}{\rho^{\prime }}$$

The volume fraction of the equivalent inclusions $V_{ef}$ is defined as the ratio between the overall volume of equivalent particles $V_{ep}$ and the volume of nanocomposite, which is expressed in Equation (11).

(11) $$V_{ef}=\frac{V_{ep}}{V_{n}}=\frac{{n\nu }_{ef}}{V_{n}}$$

Consequently, combining Equations (6), (7), (10) and (11), the volume fraction of effective particles $V_{ep}$ is related to the theoretical content of particles $V_{f}$, as shown in Equation (12).

(12) $$V_{ef}=\frac{V_{f}}{\rho^{\prime }}$$

Therefore, a novel mathematical model that incorporates the influence of the interphase, building upon the PS model, can be formulated as follows:

(13) $$\varepsilon =\varepsilon_{m}+\frac{\left(\varepsilon_{ef}-\varepsilon_{m}\right)V_{ef}}{\left(1-V_{ef}\right)\frac{\varepsilon_{ef}+2\varepsilon_{m}-V_{ef}(\varepsilon_{ef}-\varepsilon_{m})}{3\varepsilon_{m}}+V_{ef}}$$

## 3. Results and Discussion

The refined PS model is applied to estimate the dielectric permittivity of polymer-based nanocomposites containing spherical particles. To assess the reliability of the developed model, its predictions are compared with experimental data, and the key parameters ($\varepsilon_{m},\;\varepsilon_{f},\;R$) used in the calculations are summarized in Table 1. Experimental results for PVDF-BT composites [45], poly(arylene ether sulfone) (DPAES)-BT composites [46], and PVDF-BT composites [47] are selected from previously published studies. The dielectric constants ($\varepsilon_{m}$) of the polymer matrices are taken as 6.5 for PVDF [45], 3.2 for DPAES [46], and 7.5 for PVDF [47], while the permittivity of the incorporated BT nanoparticles ($\varepsilon_{f}$) is assumed to be 1240, based on reported values [48]. The average radius (R) of the BT nanoparticles used in the study is set to 50 nm, following experimental findings [49]. The experimental dielectric permittivity data for various polymer nanocomposites are utilized to determine the corresponding interphase thickness and its dielectric constant, with the computed values presented in Table 1. The results indicate that the interphase properties are strongly influenced by the combination of polymer matrix and nanoparticles. Different polymer–nanoparticle pairings lead to variations in both interphase thickness and dielectric permittivity. Despite these differences, the dielectric permittivity of the interphase consistently falls within the range defined by the permittivity of the polymer matrix ($\varepsilon_{m}$) and that of the BT nanoparticles ($\varepsilon_{f}$).

**Figure 2:**
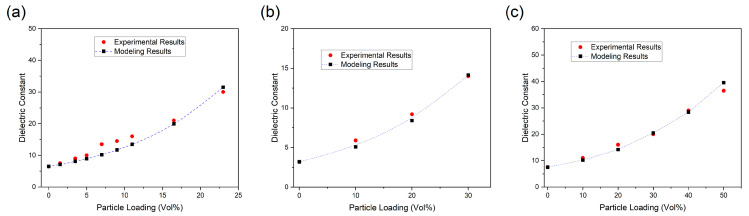
The experimental results and the modeling results of the dielectric constant of polymer-based nanocomposites: (**a**) PVDF-BT composite [45]; (**b**) DPAES-BT composite [46]; (**c**) PVDF-BT composite [47].

The predicted dielectric permittivity obtained from the developed model, along with the corresponding experimental data, is presented in Figure 2. As illustrated in the figure, the refined PS model provides highly accurate predictions for all examined samples when appropriate interphase parameters are considered, confirming its reliability in estimating the dielectric properties of BaTiO_3_–polymer nanocomposites. The strong correlation between experimental results and model predictions highlights the critical role of interphase characteristics in determining the dielectric behavior of these composites. Furthermore, disregarding interphase effects results in significant deviations, leading to inaccurate estimations of the dielectric permittivity for the reported samples. Nevertheless, it is important to acknowledge that the proposed model is developed under the assumption of spherical particles, restricting its applicability to nanocomposites with ball-shaped inclusions. Extending the refined PS model to systems containing nonspherical particles may lead to inaccuracies in prediction results.

**Figure 3:**
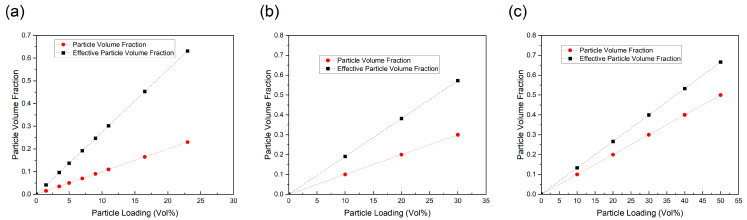
Comparison of particle volume fraction and effective particle volume fraction of polymer-based nanocomposites: (**a**) PVDF-BT composite [45]; (**b**) DPAES-BT composite [46]; (**c**) PVDF-BT composite [47].

To analyze the impact of the interphase on the effective volume concentration of nanoparticles, the volume fraction of the equivalent nanoparticles ($V_{ef}$) is computed using Equation (12) and compared with the theoretical particle content for the reported samples, as depicted in Figure 3. The results indicate that, across the entire particle loading range, the equivalent volume fraction of BaTiO_3_ nanoparticles consistently exceeds the theoretical BaTiO_3_ concentration for all experimental samples. This observation suggests that the interphase is a crucial component of the nanocomposite, significantly enhancing the effective volume fraction of nanoparticles. Consequently, the presence of the interphase plays a fundamental role in shaping the dielectric permittivity of BaTiO_3_–polymer nanocomposites.

**Figure 4:**
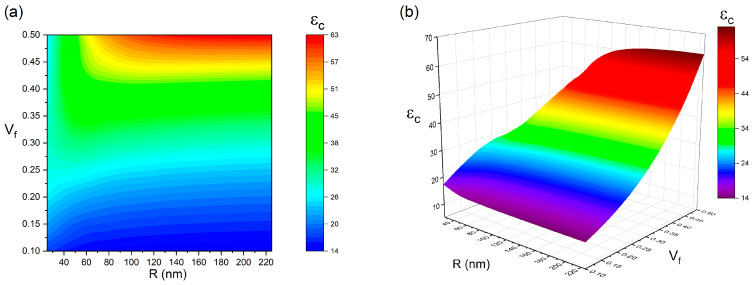
The dependence of $\varepsilon_{c}$ on the R and $V_{f}$ when $\varepsilon_{f}$ = 700, $\varepsilon_{m}$ = 10, $\varepsilon_{i}$ = 15, $R_{i}$ = 20 nm (**a**) contour plot; (**b**) 3D plot.

The size and concentration of BaTiO_3_ nanoparticles play a crucial role in determining the dielectric permittivity of BaTiO_3_–polymer nanocomposites. To systematically evaluate their effects, a parametric study was conducted. Figure 4 illustrates the relationship between the dielectric permittivity of the nanocomposite and the size and volume fraction of BaTiO_3_ nanoparticles under the conditions $R_{i}$ = 20 nm, $\varepsilon_{i}$ = 15, $\varepsilon_{f}$ = 700, and $\varepsilon_{m}$ = 10. According to the simulation results, these two factors influence the dielectric response in distinct ways. In general, the dielectric constant of the nanocomposite increases with both larger particle size and higher BaTiO_3_ loading. When the nanoparticle concentration is below 0.43, particle size has a negligible effect on dielectric permittivity. However, at BaTiO_3_ contents exceeding 0.43, a slight increase in dielectric constant is observed with increasing particle size. The maximum dielectric permittivity of 62.9 is obtained when R = 225 nm and $V_{f}$ = 50%, whereas the lowest dielectric constant ($\varepsilon_{c}$ = 13.9) is recorded at R = 225 nm with $V_{f}$ = 10%. These findings suggest that incorporating large BaTiO_3_ nanoparticles at high concentrations is a viable strategy for enhancing the dielectric performance of BaTiO_3_–polymer nanocomposites.

In polymer-based nanocomposites, the high interfacial area facilitates exchange coupling, which strengthens polarization, enhances dielectric permittivity, and improves breakdown strength. A high nanoparticle concentration with larger particle sizes contributes to an expanded interfacial area. However, due to the significant surface energy and strong van der Waals interactions, BaTiO_3_ nanoparticles have a tendency to aggregate within the polymer matrix, reducing the effective interfacial area [50]. This issue is particularly pronounced at high filler loadings and with smaller nanoparticles. Achieving a homogeneous dispersion of nanoparticles—through methods such as ultrasonication or surface modification—can mitigate this problem by maximizing the available interfacial area [51]. Moreover, a well-dispersed nanoparticle distribution leads to a uniform interphase, minimizing the permittivity gradient across the interphase region while enhancing dielectric–dielectric interactions, thereby improving the overall dielectric response.

Additionally, in nanocomposites containing spherical inclusions, a homogeneous distribution of nanoparticles prevents direct physical contact between them, thereby inhibiting the formation of conductive pathways [52]. This effect reduces the conductivity gradient across the interphase region and lowers dielectric loss while simultaneously improving breakdown strength. Consequently, optimizing nanoparticle dispersion and interfacial interactions is critical for achieving high-performance BaTiO_3_–polymer nanocomposites with superior dielectric properties.

**Figure 5:**
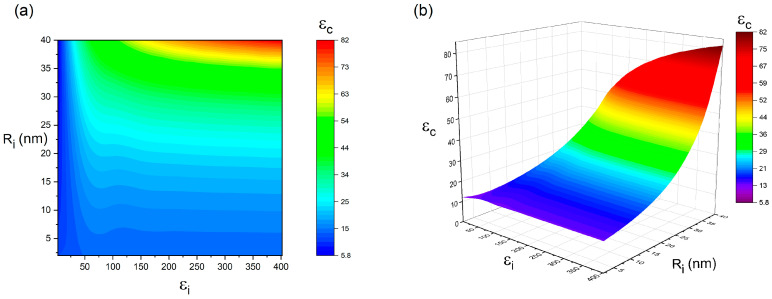
The dependence of $\varepsilon_{c}$ on the $\varepsilon_{i}$ and $R_{i}$ when $\varepsilon_{f}$ = 700, $\varepsilon_{m}$ = 10, R = 50 nm, and $V_{f}$ = 0.1 (**a**) contour plot; (**b**) 3D plot.

As previously discussed, the interphase plays a crucial role in determining the dielectric properties of spherical particle-filled nanocomposites. To further explore its impact, a parametric study was conducted to examine how interphase characteristics, including thickness ($R_{i}$) and dielectric constant ($\varepsilon_{i}$), influence the dielectric behavior of the composite. Figure 5 presents the variation in dielectric constant as a function of interphase thickness and permittivity under fixed conditions (R = 50 nm, $\varepsilon_{f}$ = 700, $\varepsilon_{m}$ = 10, and $V_{f}$ = 10%). The simulation results reveal a direct correlation between interphase attributes and composite permittivity. A thicker interphase layer combined with a higher dielectric constant enhances the dielectric response of the nanocomposite. Conversely, when the interphase is thin and its permittivity is low, the overall dielectric performance deteriorates. The highest dielectric constant ($\varepsilon_{c}$ = 82) is obtained when $R_{i}$ = 40 nm and $\varepsilon_{i}$ = 402, whereas the lowest value ($\varepsilon_{c}$ = 5.9) is recorded at $R_{i}$ = 40 nm and $\varepsilon_{i}$ = 2. These findings suggest that achieving a thick interphase with a high dielectric constant is a key strategy for optimizing the dielectric properties of BaTiO_3_–polymer nanocomposites.

Beyond improving dielectric performance, the interphase contributes to uniform nanoparticle dispersion within the polymer matrix. It also enhances interfacial interactions between ceramic nanoparticles and the surrounding polymer, reducing the likelihood of particle aggregation [53]. Furthermore, in nanocomposites incorporating conductive fillers, the interphase prevents direct particle-to-particle contact, effectively minimizing tunneling current between adjacent nanoparticles. This suppression of tunneling current results in reduced dielectric loss, enhanced dielectric strength, and an expanded compositional range, thereby improving the overall stability and performance of the composite material.

The formation of the interphase arises from both chemical and physical interactions between the nanoparticles and the surrounding polymer. For instance, the introduction of surfactant species like silane coupling agents allows a better interaction with the polymer matrix, which contributes to the formation of interphase. Due to these interactions, polymer chains adhere strongly to the surfaces of ceramic nanoparticles, leading to the development of interphase layers with distinct properties [54]. Within this region, chain mobility, conformation, and free volume differ significantly from those in the bulk polymer, ultimately influencing the dielectric characteristics of the interphase. The thickness and permittivity of this interphase zone are primarily dictated by the bonding strength between the ceramic nanoparticles and the polymer matrix, which can involve ionic, covalent, hydrogen, or van der Waals interactions, or a combination thereof [55]. Stronger bonding forces, such as ionic and covalent bonds, contribute to a thicker interphase layer. Additionally, robust interfacial interactions within the interphase enhance polarization and charge separation, leading to an interphase region with a high dielectric constant. Since a high-permittivity interphase is crucial for achieving superior dielectric performance in nanocomposites, controlling interphase thickness and properties is essential. A promising approach to tailoring these characteristics involves modifying the surface chemistry of ceramic nanoparticles to manipulate interfacial bonding strength [56].

**Figure 6:**
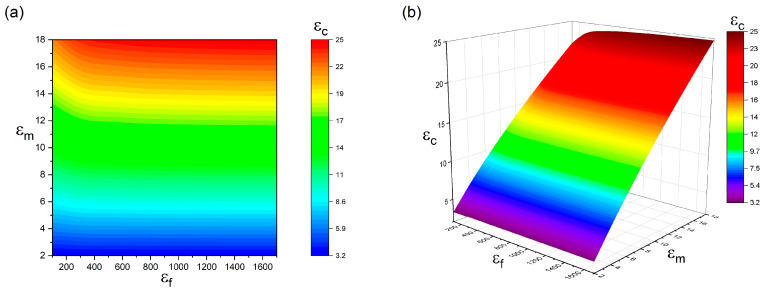
The dependence of $\varepsilon_{c}$ on the $\varepsilon_{m}$ and $\varepsilon_{f}$ when $R_{i}$ = 10 nm, R = 50 nm, $V_{f}$ = 0.1, and $\varepsilon_{i}$ = 20 (**a**) contour plot; (**b**) 3D plot.

As the fundamental components of BaTiO_3_–polymer nanocomposites, both BaTiO_3_ nanoparticles and the polymer matrix significantly impact the overall dielectric properties of the composite. Therefore, it is essential to examine how the dielectric constants of these two constituents influence the nanocomposite’s dielectric response. Based on the modified PS model, Figure 6 illustrates the dependence of the nanocomposite’s dielectric permittivity on the dielectric constants of BaTiO_3_ nanoparticles and the polymer matrix under the parameters R = 50 nm, $R_{i}$ = 10 nm, $V_{f}$ = 10%, and $\varepsilon_{i}$ = 20. The results indicate that increasing both $\varepsilon_{m}$ (polymer matrix permittivity) and $\varepsilon_{f}$ (BaTiO_3_ nanoparticle permittivity) leads to an enhancement in the composite’s dielectric properties. Conversely, lower values of $\varepsilon_{m}$ and $\varepsilon_{f}$ result in reduced dielectric performance. Additionally, the modeling results suggest that the polymer matrix has a more pronounced effect on the overall dielectric constant than the BaTiO_3_ nanoparticles, likely due to the limited particle concentration. The highest dielectric constant ($\varepsilon_{c}$ = 24.8) is achieved when $\varepsilon_{f}$ = 1700 and $\varepsilon_{m}$ = 18, whereas a low-permittivity matrix ($\varepsilon_{m}$ = 2) combined with a small nanoparticle permittivity ($\varepsilon_{f}$ = 100) produces a composite with a significantly reduced dielectric constant ($\varepsilon_{c}$ = 3.28). These findings highlight that using both a high-permittivity polymer matrix and ceramic fillers is an effective strategy for improving the dielectric performance of BaTiO_3_–polymer nanocomposites.

Polymer-based nanocomposites offer advantages such as cost-effectiveness and ease of processing, making them highly desirable for dielectric applications [57]. The choice of polymer used as the matrix plays a crucial role in determining the overall dielectric permittivity of the composite. Studies have shown that enhancements in the polymer matrix’s dielectric constant contribute significantly to the overall improvement of nanoparticle-filled nanocomposites. Specifically, polar polymers such as PVDF exhibit high intrinsic permittivity due to their molecular dipole moments, which facilitate an increase in the dielectric performance of BaTiO_3_–polymer nanocomposites.

## 4. Conclusions

A modified PS model was established to predict the dielectric permittivity of polymer nanocomposites filled with spherical particles, incorporating the effects of the interphase. By appropriately selecting interphase parameters, the model demonstrates strong consistency with experimental data. Additionally, the findings reveal that neglecting interphase contributions leads to an underestimation of the effective nanoparticle concentration. Moreover, parameter analysis indicates that the dielectric permittivity of nanocomposites is influenced by multiple factors. To achieve high dielectric performance, both the polymer matrix and embedded nanoparticles should possess high permittivity. A thick interphase layer with a high dielectric constant enhances the dielectric response of the composite, whereas a thin interphase with low permittivity negatively affects its performance. Furthermore, increasing the nanoparticle concentration and using larger particles improves the overall dielectric properties, while a low filler content with small nanoparticles results in reduced permittivity. It is worth noting that this modeling approach is applicable not only to BaTiO_3_–polymer nanocomposites but also to other polymer-based composites incorporating spherical inclusions. As a result, the developed model is expected to serve as a valuable tool for designing advanced nanocomposites with optimized dielectric properties.

## Figures and Tables

**Table 1:** The parameters of polymer nanocomposites from reported studies and the calculated results based on the developed PS model.

Samples	$\varepsilon_{m}$	$\varepsilon_{f}$	$R_{i}(nm)$	R(nm)	$\varepsilon_{i}$
PVDF-BT composite [45]	6.5	1240	20	50	23.7
DPAES-BT composite [46]	3.2	1240	12	50	8
PVDF-BT composite [47]	7.5	1240	5	50	7.8

## Data Availability

The original contributions presented in this study are included in the article. Further inquiries can be directed to the corresponding authors.
